# Computational Fluid Dynamics (CFD) Model for Analysing the Role of Shear Stress in Angiogenesis in Rheumatoid Arthritis

**DOI:** 10.3390/ijms24097886

**Published:** 2023-04-26

**Authors:** Malaika K. Motlana, Malebogo N. Ngoepe

**Affiliations:** 1 Department of Mechanical Engineering, University of Cape Town, Rondebosch, Cape Town 7701, South Africa; 2 Centre for Research in Computational and Applied Mechanics (CERECAM), University of Cape Town, Rondebosch, Cape Town 7701, South Africa

**Keywords:** rheumatoid arthritis (RA), vascular endothelial growth factor (VEGF), angiogenesis, wall shear stress, blood vessels, pathogenesis, computational fluid dynamics (CFD), CFD model

## Abstract

Rheumatoid arthritis (RA) is an autoimmune disease characterised by an attack on healthy cells in the joints. Blood flow and wall shear stress are crucial in angiogenesis, contributing to RA’s pathogenesis. Vascular endothelial growth factor (VEGF) regulates angiogenesis, and shear stress is a surrogate for VEGF in this study. Our objective was to determine how shear stress correlates with the location of new blood vessels and RA progression. To this end, two models were developed using computational fluid dynamics (CFD). The first model added new blood vessels based on shear stress thresholds, while the second model examined the entire blood vessel network. All the geometries were based on a micrograph of RA blood vessels. New blood vessel branches formed in low shear regions (0.840–1.260 Pa). This wall-shear-stress overlap region at the junctions was evident in all the models. The results were verified quantitatively and qualitatively. Our findings point to a relationship between the development of new blood vessels in RA, the magnitude of wall shear stress and the expression of VEGF.

## 1. Introduction

Rheumatoid arthritis (RA) is a chronic, autoimmune disease in which the body’s immune system attacks healthy tissue cells found in the lining of the joints [1,2,3]. RA impacts this lining, known as the synovium or synovial membrane. Owing to the involvement of many different variables, the pathophysiology of RA is still not fully understood [4]. However, angiogenesis is crucial to the progression of RA and results in the formation of new blood vessels [5,6,7,8]. Under physiological conditions, angiogenesis is governed by a complex, balanced network of chemical and mechanical cues, which maintain vital physiological functions [9]. In pathological cases such as RA, the altered environment and unbalanced angiogenetic processes contribute to disease progression [9,10].

Various sources have reported increased angiogenesis in RA patients, which can be detected at the point of clinical diagnosis [6]. RA produces a radically altered synovial composition with a reduced viscosity [11]. This enables pannus formation in the affected joint [3,10,12,13,14,15,16]. The environment in the pannus is hypoxic and inflammatory, necessitating increased angiogenesis to supply oxygen and nutrients [6,14,17]. Another view is that angiogenesis, a process natural to the body, drives RA through the development and maintenance of the pannus in an environment that upregulates vascular endothelial growth factor (VEGF) [7,10,12]. Although different, these perspectives elucidate the complexity of the disease and support the idea that RA angiogenesis arises from a misbalance between stimulating and inhibiting factors [10].

VEGF is essential to angiogenesis under physiological and pathological conditions [18,19,20,21]. Clinically, patients with RA present with increased VEGF levels in both serum and synovial fluid [22,23,24]. The role of VEGF-related genes has also been explored in these patients [7,23,25,26,27,28]. Elevated VEGF levels correlate with markers of inflammation and RA activity, such as increased C-reactive protein and an increase in swollen joints [29,30,31]. A wide range of factors, including hypoxia, hormones and signalling networks, all influence the expression of VEGF [32,33,34,35,36]. In addition, mechanical variables such as shear stress contribute to VEGF expression and angiogenesis [37].

Unlike genetics and chemical factors, the relationships between mechanical factors, angiogenesis, VEGF and RA have been less explored [37]. Shear stress has been shown to influence angiogenesis significantly, and the extracellular matrix’s mechanical behaviour has been analysed [38,39,40,41,42]. From a fluid mechanics perspective, shear stress is pivotal when considering blood flow. In blood vessels, localised blood flow patterns influence a range of stress and stretch measures, including the wall shear stress experienced by blood vessel wall components [43,44,45]. These forces arise from many haemodynamic variables, including the pulsatile nature of blood flow and pressure [46]. Shear stress in the blood vessels can affect morphology, organisation of the endothelial cytoskeleton, the functioning of ion channels and gene expression within endothelial cells [47,48,49]. Given the wide-ranging impact, the effects of wall shear stress on VEGF and angiogenesis are varied [46].

A shear stress threshold of 1 Pa was found to trigger angiogenic sprouting in endothelial cells, which could then penetrate the underlying matrix [50]. Blood fluid shear stress has also been linked to the upregulation of VEGF gene expression [51]. One study hypothesised that shear stress generated by considerable differences between capillary, venous and arterial blood flow might influence the observed differences in VEGF expression [52]. In a murine model, VEGF was expressed exclusively by arterial endothelial cells. When laminar arterial shear stress was applied to human umbilical vein endothelial cells (HUVEC), the expression of VEGF increased, but the exact mechanism remained unclear. A more recent microfluidic study demonstrated that departure from the stabilised state, either in shear stress or VEGF concentration, led to neovascularisation [53]. A recent study estimated shear stress in endothelial cells in regions of neovascularisation [54]. The authors highlighted that values above 0.1 Pa were physiologically relevant. The expression of VEGF increased with both pulsatile flow and laminar shear stress [55,56]. While it is clear that shear stress and other mechanical variables play an essential role in VEGF regulation and angiogenesis, the application of these findings to RA remains limited [52].

Although many studies of angiogenesis and blood flow have been conducted in vitro, computational fluid dynamics (CFD) could augment these approaches. Significant advances in computing technology have improved the approximation of solutions for the Navier–Stokes equations, which describe fluid flow in a wide range of contexts [57,58,59,60,61,62,63,64,65,66,67]. As a result, CFD models have been developed for a plethora of applications, including biofluid flows [68,69,70,71]. The solvers compute pressure and velocity results, which can then be used to calculate quantities such as shear stress. In biofluid flows, CFD models provide a potential avenue for quantifying and exploring various mechanical variables, such as shear stress, in blood vessels. Countless cardiovascular CFD models have enabled the quantification of variables which would prove challenging to measure in other settings [59,72]. For example, a CFD angiogenesis model estimated shear stresses experienced by endothelial tip cells [54]. A limitation of CFD is computational cost, with very sophisticated models requiring significant computing resources and time [73]. Model developers must consider how different aspects of the model may be represented and which assumptions could enable reasonable simplification. Kretschmer et al. demonstrate that angiogenesis relies on mechanical and chemical factors [37]. They further argue that chemical cues can be translated into mechanical signals and vice versa. Together with Leblonde et al., they highlight that VEGF is one of the most critical drivers of RA angiogenesis and that VEGF plays a vital role in the mechanics of angiogenesis [3]. In a simplified CFD angiogenesis model, shear stress could be a proxy for VEGF [52,54,74].

This study explores the role of shear stress, as a surrogate for VEGF, in developing new blood vessels in RA. Using CFD, the study examines how shear stress emanating from the blood flow coincides with the location of new blood vessels and the progression of RA. This is achieved by analysing patterns of blood flow variability that may relate to where new blood vessels emerge within the blood vessel network. These findings are compared to a micrograph of blood vessel networks in RA. Understanding these patterns may be beneficial in gaining insight into the significance of the magnitude of shear stress and, by implication, the expression of VEGF.

## 2. Results

The results section begins with a brief note on mesh independence. The geometries from Section 2.2 were discretised to enable the numerical solution of the governing equations, and this process resulted in the development of a mesh. Greater detail regarding mesh independence is given in Section 2.1, followed by velocity and shear stress results for Models 1 and 2.

### 2.1. Mesh Independence

**Figure 1:**
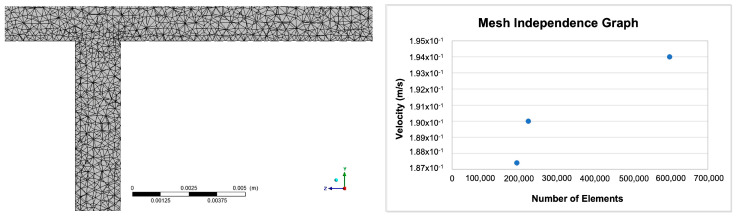
An image of the mesh generated from the original geometry and a graph illustrating grid independence for the model.

For a fluid flow solution, sufficient refinement must be achieved to ensure that the mesh does not have a negative impact on the numerical solution. A solution computed for an adequately refined mesh is considered mesh/grid independent. A less than 2% error was deemed an acceptable value for mesh independence in this study. The element size for the mesh independence was determined by modelling a single blood vessel represented by a solid cylinder of radius 0.001 m and length of 0.1 m. The mesh was generated with an initial element size of 0.0000334 m, and the blood velocity was set to 0.19 m/s at the inlets. The process was repeated twice, decreasing the element size in each subsequent run. Given that wall shear stress is calculated from velocity (and CFD computes velocity and pressure fields), velocities at the outlet of the flow field were compared, and the error (1.41%) was found to be sufficiently small for an element size of 0.0000334 m. Both the mesh and the mesh independence graph are illustrated in Figure 1.

### 2.2. Model 1

Due to the wall shear stress being significantly higher than that prescribed for physiological arterial shear, the blood velocity was decreased from 0.19 m/s in the mesh independence study to 0.09 m/s in Models 1 and 2. The velocity was therefore reduced according to the Haagen–Poiseuille equation until the arterial shear range was satisfied.

**Figure 2:**
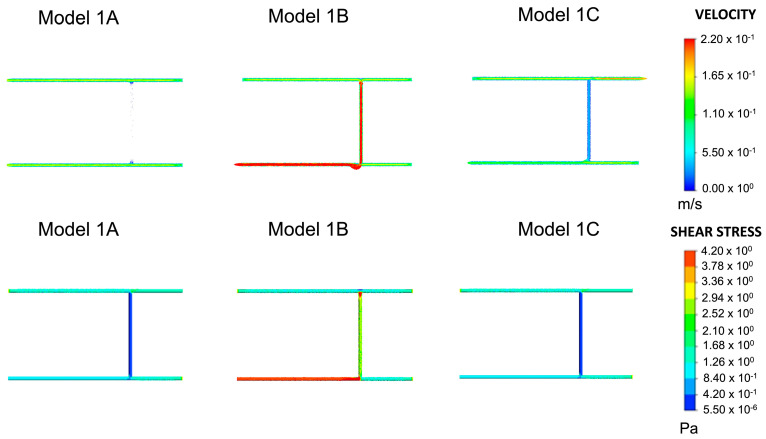
Velocity and shear stress results for Models 1A–1C. This was the same model with variations on inlets and outlets, as shown in Figure 2. Given the plausible velocity and shear stress ranges, Model 1C was chosen as the baseline model for all subsequent variations of Model 1.

The shear stress in wall 2 of Model 1A, shown in Figure 2, is outside the previously defined range. The velocity vectors indicate almost no flow within wall 2. The velocity was in the range of 0.00–0.055 m/s. This is a non-physiological finding because blood in the body constantly moves in the circulatory system. The position and number of inlets and outlets in Models 1B and 1C were adjusted to evaluate their effect on velocity. Compared to Model 1A, Model 1B showed a significant increase in the velocities (>0.22 m/s) of blood vessels 1 and 2. Parts of wall 1 had shear stresses greater than 4.2 Pa. Both wall shear stress and velocity lay outside the range of blood flow conditions in this model. Except for wall 2, which experienced the same shear stresses as Model 1A, Model 1C achieved plausible physiological velocity and wall shear stress values. Therefore, subsequent models were built from Model 1C. Junction 1 had a wall shear stress range of 0.420–1.260 Pa, and junction 2 had a range of 0.840–2.520 Pa. The two ranges intersected between 0.840–1.260 Pa. Based on this range, additional blood vessels were added in subsequent models. The junction overlap region for Model 1, shown in Table 1, is defined as the intersection between the wall shear stress range at all the junctions within a single model.

**Figure 3:**
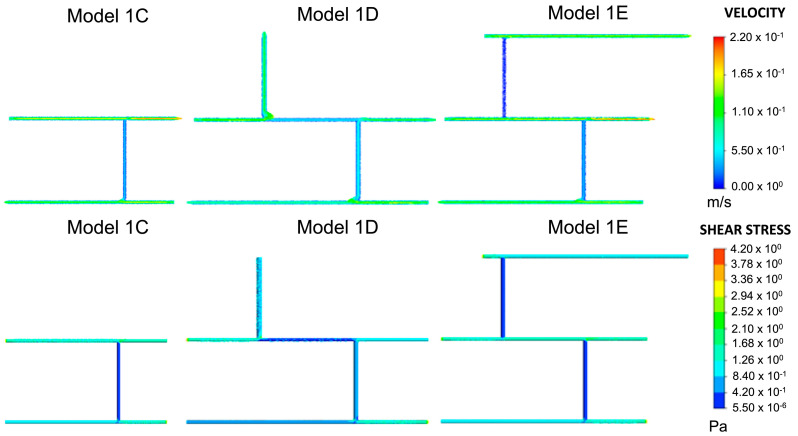
Velocity and shear stress results for Models 1C–1E. This was the same model with variations on inlets and outlets, as shown in Figure 2. Given the plausible velocity and shear stress ranges, Model 1C was chosen as the baseline model for all subsequent variations of Model 1.

As previously described, models were built from Model 1C, as shown in Figure 3. Velocities in Model 1D fell predominantly within the desired range of 0.049–0.19 m/s. In regions where the velocity increased, the wall shear stress also increased, as predicted by the Hagen–Poiseuille equation. This directly proportional relationship also held where the velocity decreased. The overlap between junctions 1, 2, and 3 was 0.840–1.260 Pa. The next blood vessel was added to the network at wall 4, where the shear stress fell within this range. In Model 1E, the velocity and wall shear stress were lower in blood vessel walls 2 and 4 than in Model 1D. There were no considerable changes elsewhere in the blood vessel network. Consequently, the wall shear stress overlap at junctions 1–4 remained between 0.840–1.260 Pa.

**Figure 4:**
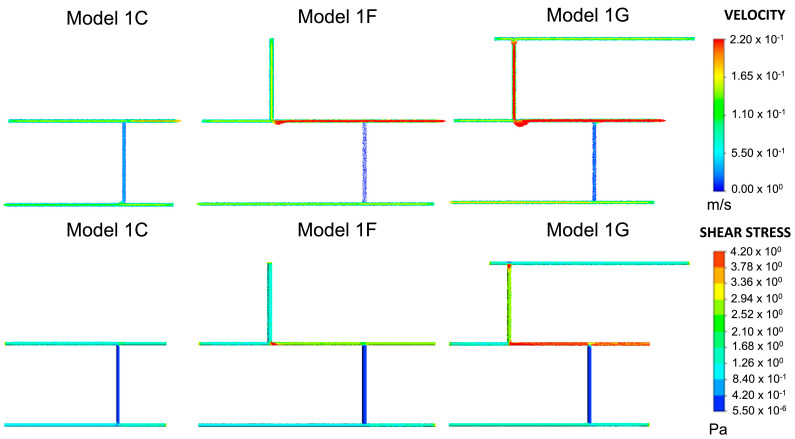
Model 1F and Model 1G are derived from Model 1C. These are similar to Model 1D and Model 1E, respectively, with the exception of inlet/outlet arrangements.

Models 1F and 1G, shown in Figure 4, were used to analyse how the position and number of inlets and outlets would affect the junction overlap region. Models 1D and 1F had the same wall shear stress at junction 1. In Model 1F, junctions 2 and 3 had higher shear stress ranges than in Model 1D. Hence, no overlap occurred. The overlap region seen in Model 1G was higher than the overlap region seen in the other models, between 1.260–1.680 Pa. Therefore, it was regarded as an outlier. Compared to Model 1E, the shear stress at the junctions was higher and exceeded the limit for wall shear stress and velocity in wall 3.

### 2.3. Model 2

**Figure 5:**
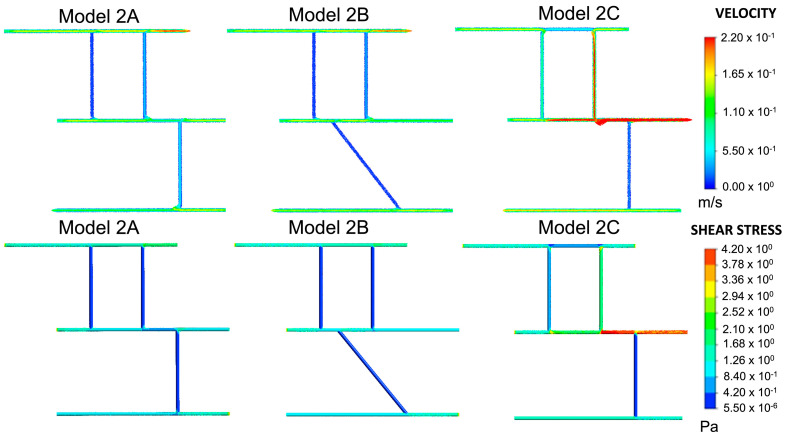
Velocity and shear stress plots for Models 2A, 2B and 2C. All three models represent the complete network.

For most of the vessels in Model 2A, shown in Figure 5, the wall shear stress lay within the range of the physiological arterial shear, and the velocity followed a similar pattern. Blood vessels 2, 3, and 5 were the exceptions to this observation. The results of Model 2A (Figure 5) were compared to Model 1E (Figure 3) as they were similar in shape. Although Model 2A had six blood vessels compared to five, the main difference between the two models lay in the direction of the highest-numbered blood vessel. In Model 2A, this blood vessel developed towards the left, whereas in Model 1E, it developed towards the right. Model 2A had the same wall shear as Model 1E at junction 1 and wall 1. The shear stress in wall 2 was also the same for both models. The wall shear for junctions 2, 5, and 6 in Model 2A, which were in a similar position to junctions 2, 3 and 4 in Model 1E, had similar values. Ultimately, the junction overlap region for Models 2A and 1E was identical. Model 2B investigated whether placing a blood vessel at an acute or obtuse angle would affect the wall shear and velocity. In blood vessel 2, the difference in the velocity was marginal compared to Model 2A; the wall shear and junction overlap remained the same. Thus, angling a blood vessel has little to no effect on the wall shear and velocity. Outlet 5, in Model 2A, was changed to inlet 5 in Model 2C (Figure 10). The velocity of blood vessels 1 and 4 increased as a result. Consequently, the wall shear was also higher in that region. The velocity and wall shear stress in blood vessels 2 and 3 were outside the already defined range of 0.049–0.19 m/s and 0.6–4 Pa, respectively. The junction overlap regions for Model 2 are shown in Table 2.

### 2.4. Model Verification

**Figure 6:**
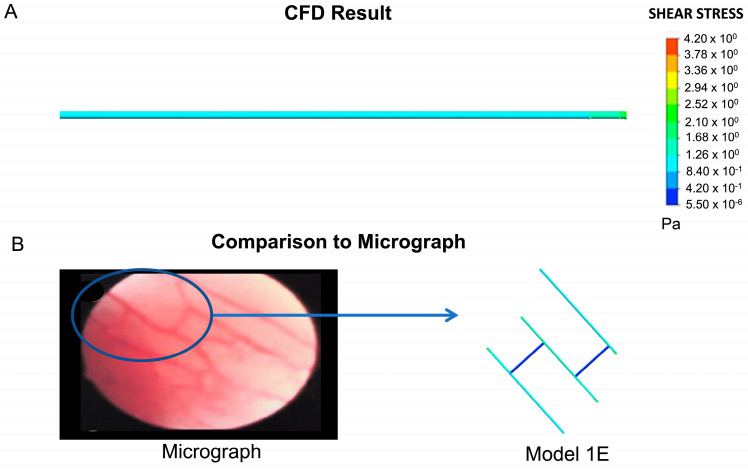
Quantitative and qualitative verification of the computational model. (**A**) Shear stress plot for a straight pipe based on the same parameters as those employed in Models 1 and 2. (**B**) Comparison between the geometry that emerged from Model 1 (Model 1E) and the original micrograph that was used to inform the initial model (permission obtained from RightsLink/Elsevier) [75].

The shear stress for the straight vessel was computed as 1.26 Pa, as shown in Figure 6A. On the basis of the analytical Hagen–Poiseuille formulation, we also calculated shear stress as 1.26 Pa. This verifies our result quantitatively. Qualitatively, Models 1E and 1G are very similar to the image shown in the micrograph (Figure 6B). Both models emerge from the initial geometry and emanate from the shear stress thresholds described above.

## 3. Discussion

The objective of this study was to determine how blood flow influences angiogenesis in RA. Shear stress, a mechanical variable dependent on flow, was used as a surrogate for VEGF in determining the location of the new blood vessels. To achieve our objectives, two models of blood vessel networks in RA were built using CFD to analyse whether blood vessels would develop in low or high shear stress regions. Although several CFD studies have already been used to examine shear stress values in angiogenesis broadly, these have yet to be extended to RA [54,76]. Even in the in vitro angiogenesis studies, the exact role of wall shear stress remains controversial, with similar values having been shown to enhance and attenuate angiogenetic sprouting [46]. In this discussion, we consider our results in the context of other computational models and discuss the link between shear stress and VEGF.

The results of our two models, which are specific to RA, indicate that new blood vessels will form in areas of relatively low shear stress. Specifically, new branches formed between 0.840–1.260 Pa, which is in the lower half of the 0.6–4.20 Pa range. The overlap region for the wall shear stress at the junctions was the same for all models in our study, indicating a relationship between the emergence of new blood vessels and the magnitude of the wall shear stress. Our shear stress values are in the same order of magnitude as those in other computational studies that examined the effect of side branches [54,76,77,78]. In their CFD study on shear stress values along endothelial tip cells at the end of the capillary sprout in angiogenesis, Hu et al. considered a wall shear stress above 0.1 Pa to be physiologically relevant [54]. They found that tip cell shear stresses ranged from 0.019–0.465 Pa, and three out of eight cases achieved values above 0.1 Pa. Stapor et al. examined wall shear stress in angiogenetic capillary sprouts using CFD [76]. They found a local maximum wall shear stress value of 1.4 Pa at the base of a sprout with a non-permeable vessel wall. For larger blood vessels, such as the coronary arteries modelled by Wellnhofer et al. in a CFD model, a median wall shear stress value of 2.54 Pa was reported for steady state simulations with side branches [77]. In a fluid-structure interaction model using CFD to solve the fluid part, Ngoepe et al. found that for models with different side branch geometries, the peak wall shear stress varied from 0.7–2.3 Pa in arterial to venous anastomosis models [78]. The values from literature show that our shear stress values fall within the range of other CFD studies that include side branches. Wellnhofer et al. found that including side branches was necessary for wall shear stress estimation [77]. In particular, they found that the spread and distribution of wall shear stress, particularly for high and low values, was increased by including side branches. In our models, some blood vessels did not meet the arterial velocity or wall shear conditions, particularly the vertically orientated blood vessels. Wall shear stress in these vessels generally ranged between 5.5 × 10^−6^–0.42 Pa, and the velocity was between 0.00–0.055 m/s. In their CFD model, Wellnhofer et al. found that very low wall shear stresses (i.e., less than 0.4 Pa) occurred in aneurysmatic coronary artery disease cases. In addition to these in silico findings, Galie et al. found that a shear stress threshold of 1 Pa triggered angiogenetic sprouting in an in vitro study [50].

Given that shear stress was used as a surrogate for VEGF, it is important to link our computed shear rate values to experimental observations of VEGF. In a microfluidic study examining the combined effect of shear stress and VEGF on neovascularisation, Zhao et al. found that shear stress plays a dominant role when VEGF is sufficient [53]. They found initiating neovascularisation under 1.5 Pa difficult, even with enough VEGF. Their threshold value is slightly higher than our maximum value for branch formation (1.260 Pa). Fey et al. examined the role of VEGF and shear stress on podosomes, which play a pivotal role in cell motility and are important for angiogenesis in endothelial cells [79]. In the absence of VEGF, changes in shear stress did not affect cell density, but higher shear stresses resulted in less podosome activity. When considering shear stress and VEGF together, it was found that high shear stress (1 Pa) increased podosome activity when there was sufficient VEGF in the system. This high shear stress value falls within our predicted angiogenetic range. Russo et al. considered how altering shear stress may change growth factor gene expression in endothelial cells [47]. A reduction from a physiological to pathological shear stress value (1.2 Pa to 0.4 Pa) increased VEGF gene expression. The pathological value fell outside our angiogenetic range, but direct comparison is somewhat challenging given that our model could not account for gene expression. Overall, our computed results fell in a range that supports VEGF expression and angiogenesis.

Our results demonstrate the importance of shear stress in RA angiogenesis and provide a tool for exploring the influence of haemodynamics. In cases where it is challenging to locate VEGF expression in newly developing blood vessels in RA, a haemodynamic simulation could map shear stress in the vascular network. Using wall shear stress as a surrogate for VEGF, researchers could identify parts of a blood vessel network where VEGF expression is likely to be highest. The application of this knowledge could contribute to the development of anti-VEGF biological therapies that inhibit expression. Some have even suggested that limiting angiogenesis by blocking the blood supply in the pannus may benefit patients [3]. Challenges to many therapeutic approaches would arise from patient variability. The main limitations of our work are the exclusion of chemical factors and the simplification of the blood flow. If developed further, the CFD model could account for patient-specificity factors such as VEGF and hypoxia, thereby enabling a coupled consideration of some of the most important variables for RA angiogenesis [3]. Other assumptions that should be revisited include modelling the blood flow as steady and laminar, with blood behaving as a Newtonian fluid. Furthermore, the role of the distensibility of the newly formed vessels should be explored.

## 4. Materials and Methods

The methods below describe the CFD simulations and blood vessel configurations used to analyse the relationship between the blood flow, shear stress, and the growth of new blood vessels. The experimental study employs steady-state conditions and makes several simplifying assumptions.

### 4.1. Fluid Flow Simulations

ANSYS Fluent Version 20.2.0 (ANSYS, Lebanon, NH, USA), a computational fluid dynamics simulation software, was used to model the blood flow in the respective geometries. ANSYS Fluent solves the Navier–Stokes equations by discretising the partial differential equations that govern the flow using the finite volume method (FVM),

(1) ∇·U=0,

(2) $$\rho \frac{\partial U}{\partial t}+\rho U+\nabla U+\nabla P=\;\mu \nabla^{2}U,$$

where *U* is velocity, ρ is the fluid density, μ is the dynamic fluid viscosity, *t* is time and P is pressure.

#### Boundary Conditions and Assumptions

Although a fair amount of pulsatility is experienced in blood vessels, steady-state conditions were applied. It has been shown that for more extended periods, such as when new vessel growth takes place, the baseline steady-state effect dominates the mechanical environment sensed by the cells [80]. In their CFD study, Wellnhofer et al. found that steady-state simulations were appropriate for a time-averaged wall shear stress [77]. Blood was assumed to be a Newtonian fluid with a density ρ = 1060 kg/m^3^ and a constant viscosity of μ = 0.0035 kg/m·s.

Even though angiogenesis is characterised by the formation of microvessels in the synovium [81], we were interested in exploring the role of arterial shear stresses as these have been implicated in marked VEGF expression [52]. As such, we had to balance two competing priorities: making the vessels sufficiently small while achieving arterial shear stress. The Haagen–Poiseuille equation was used to determine the maximum blood flow velocity and the vessel diameter that would result in shear stress inside the physiological arterial blood flow range (0.6–4 Pa) [52]. The arterial blood flow velocity was restricted to 0.049–0.19 m/s [82]. The blood vessel needed a diameter ranging from 0.1–10 mm [83]. All this information informed the selection of the blood vessel diameter and the maximum velocity. The blood vessels were assumed to be cylindrical and rigid, and a no-slip boundary condition was applied to the walls.

### 4.2. Geometries and Modelling Approach

The geometries are based on a micrograph of small blood vessels in the knee joint of an RA patient, which is presented in a study by Cañete et al. [75]. The vessels had a straight, branching pattern that is characteristic of RA. Although the exact dimensions of the vessels were not given, the micrographs were obtained using a 1.9 mm diameter or 2.7 mm diameter arthroscope.

**Figure 7:**
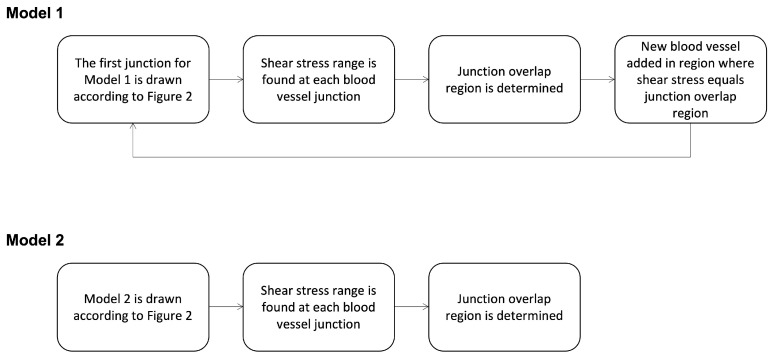
Two different approaches to developing the blood vessel network. Model 1 progressively adds vessels based on thresholds, while Model 2 begins with the entire vessel network.

Two different approaches, shown in Figure 7, were taken to develop models of blood vessel networks. The first approach (Model 1), shown in Figure 7, progressively added vessels based on shear stress thresholds. Given the strong link between shear stress and angiogenesis, we sought to find shear stress values that might support the development of new blood vessels. The process for determining the thresholds is described in the following paragraph and the threshold values are shown in Table 1 and Table 2. The second approach (Model 2), also shown in Figure 7, modelled the complete vessel network as a starting point. Both models were used to analyse the relationship between wall shear stress and the development or positioning of new blood vessels. These examined how low shear and high shear regions influence blood vessel formation.

**Figure 8:**
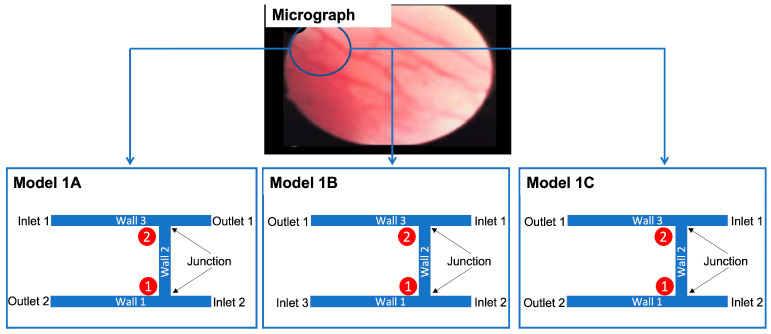
Development of models from realistic geometry for Model 1. The geometries for the initial geometry are derived from a micrograph of blood vessels in RA (permission obtained from RightsLink/Elsevier) [75]. The portion in the solid circle informs (B) the starting point for Model 1. The different variations (Model 1A, Model 1B and Model 1C) arise from rearranging inlets and outlets.

The first approach, Model 1, began with a junction comprising two horizontal vessels connected by one vertical vessel. A three-vessel representation, comprising solid cylinders of radius 0.001 m, models a subsection of the blood vessels as depicted in Models 1A, 1B and 1C in Figure 8. Shear stress and velocity values were computed for these three different arrangements, where the positions of the inlets and outlets were varied. Model 1C was chosen as the final starter model as it achieved plausible physiological shear stresses and blood flow velocities.

**Figure 9:**
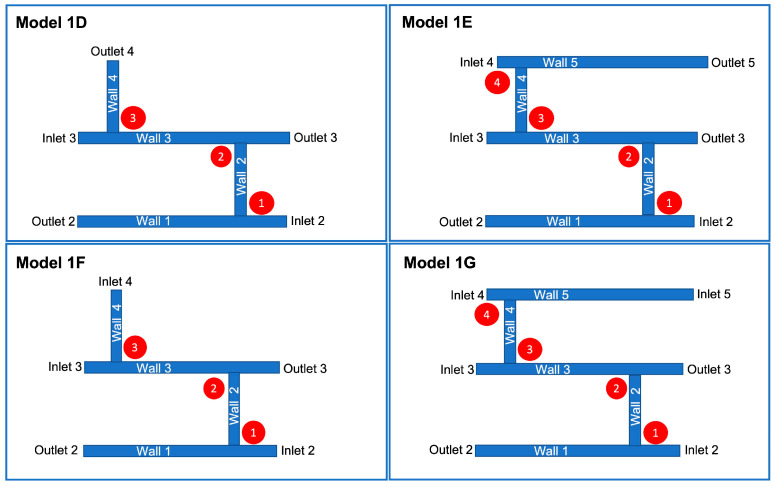
Geometries which emerge from vessels added to Model 1C. Models 1D and 1F are similar, except for inlet/outlet 4. Similarly, Models 1E and 1G differ only in so far as inlet/outlet 5 is concerned.

As described in Figure 8, subsequent geometries emerged from Model 1C, which determined where new blood vessels would form based on the range of shear stress values observed at junctions one and two. Shear stress was also calculated for wall three. A search for portions that achieved the range observed at junctions one and two was conducted. The part(s) of the wall that met this threshold were deemed capable of angiogenesis, and a vertical cylinder of length 0.05 m was constructed at these respective locations. This process was repeated twice, and the geometries which emerged from this process (Model 1D and Model 1E) are shown in Figure 9. Models 1F and 1G, also shown in Figure 9 and based on Models 1D and E, respectively, were included to analyse the effect of changing the numbers of inlets and outlets. The corresponding shear stress and velocity results for all the geometries are presented in the results section.

**Figure 10:**
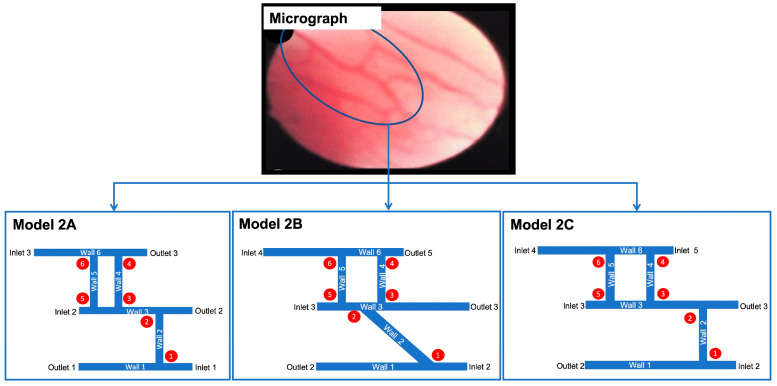
Development of models from realistic geometry for Model 2. The geometries for the initial geometry are derived from a micrograph of blood vessels in RA (permission obtained from RightsLink/Elsevier) [75]. The portion in the solid is the complete geometry for Model 2. The different variations (Model 2A, Model 2B and Model 2C) arise from rearranging inlets and outlets and placing one of the vessels at an angle.

Figure 10 illustrates the designs for the second approach, Model 2. As described in Figure 7, this approach assumes the entire network as its starting point and examines shear stresses at the junctions of blood vessels. Variations on this basic model include changing the boundary conditions and placing one of the vertical vessels at an angle. These alterations are presented in Models 2A, 2B and 2C, shown in Figure 10 in the results section. The corresponding shear stress and velocity results for all the geometries are presented in the results section.

### 4.3. Verification of Results

We verify our results quantitatively and qualitatively. For the former, we model blood flow through a straight pipe of radius 0.001 m, equivalent to the side branches in our other models. In this simple model, we use the same blood parameters and boundary conditions as those for Models 1 and 2. Once we have run the model, we compare the shear stress result to that calculated using the Hagen–Poiseuille formulation. This analytical solution enables us to calculate velocity and shear stress in straight pipes. The shear stress equation is given as

(3) $$\tau =\frac{4\mu Q}{\pi r^{3}}$$

where τ is the shear stress, Q is the volumetric flow rate and r is the pipe radius. Qualitatively, we compare the final configuration for Model 1 with the original image that informed the initial model [75].

## 5. Conclusions

The findings of this study highlight the role of haemodynamics and wall shear stress in angiogenesis in rheumatoid arthritis. Specifically, we observed that new blood vessels are likely to develop in regions of low wall shear stress (0.840–1.260 Pa) in a computational fluid dynamics model. This highlights the role of mechanical factors in RA angiogenesis and provides a tool for further exploration of this phenomenon. Other angiogenesis-driven diseases, such as cancer, may also benefit from a similar modelling approach.

## Figures and Tables

**Table 1:** Wall shear stress and velocity ranges, and wall shear stress junction overlap regions at different locations in the blood vessel network for different variations of Model 1.

Model	Location of Wall Shear Stress	Wall Shear Stress Range (Pa)	Junction Overlap Region (Pa)	Velocity Range (m/s)
1C	Junction 1	0.420–1.260	0.840–1.260	0.055–0.11
	Junction 2	0.840–2.520		0.055–0.22
	Wall 1	0.840–1.680		0.11–0.165
	Wall 2	0.0000055–0.420		0.00–0.055
	Wall 3	0.840–2.10		0.055–0.22
1D	Junction 1	0.420–1.260	0.840–1.260	0.055–0.165
	Junction 2	0.840–1.680		0.055–0.11
	Junction 3	0.840–2.10		0.11–0.165
	Wall 1	0.420–1.680		0.11–0.165
	Wall 2	0.420–0.840		0.00–0.055
	Wall 3	0.0000055–1.680		0.055–0.165
	Wall 4	0.420–1.260		0.055–0.11
1E	Junction 1	0.420–1.260	0.840–1.260	0.055–0.165
	Junction 2	0.840–1.680		0.00–0.055
	Junction 3	0.840–2.10		0.055–0.11
	Junction 4	0.420–1.680		0.055–0.11
	Wall 1	0.840–1.260		0.11–0.165
	Wall 2	0.0000055–0.420		0.00–0.055
	Wall 3	0.840–2.10		0.11–0.22
	Wall 4	0.0000055–0.420		0.00–0.055
	Wall 5	0.840–1.680		0.11–0.165
1F	Junction 1	0.420–1.260	No overlap	0.00–0.055
	Junction 2	0.840–2.10		0.11–0.165
	Junction 3	1.260–4.20		0.11–0.165
	Wall 1	0.840–1.260		0.11–0.165
	Wall 2	0.0000055–0.420		0.00–0.055
	Wall 3	0.840–2.950		0.11–0.22
	Wall 4	0.840–1.680		0.11–0.165
1G	Junction 1	0.420–1.680	1.260–1.680	0.00–0.055
	Junction 2	0.840–4.20		0.055–0.11
	Junction 3	0.840–4.20		0.165–0.22
	Junction 4	1.260–4.20		0.11–0.22
	Wall 1	0.840–1.680		0.11–0.22
	Wall 2	0.0000055–0.420		0.00–0.055
	Wall 3	0.840–4.20		0.11–0.22
	Wall 4	1.680–2.940		0.165–0.22
	Wall 5	0.840–1.680		0.11–0.165

**Table 2:** Wall shear stress and velocity ranges, and wall shear stress junction overlap regions at different locations in the blood vessel network for different variations of Model 2.

Model	Location of Wall Shear Stress	Wall Shear Stress Range (Pa)	Junction Overlap Region (Pa)	Velocity Range (m/s)
2A	Junction 1	0.420–1.260	0.840–1.260	0.055–0.11
	Junction 2	0.840–2.10		0.055–0.11
	Junction 3	0.420–1.260		0.055–0.11
	Junction 4	0.840–2.520		0.055–0.11
	Junction 5	0.420–1.260		0.00–0.11
	Junction 6	0.840–2.10		0.00–0.11
	Wall 1	0.840–1.260		0.11–0.165
	Wall 2	0.0000055–0.420		0.00–0.055
	Wall 3	0.420–1.680		0.11–0.165
	Wall 4	0.0000055–0.420		0.00–0.055
	Wall 5	0.0000055–0.420		0.00–0.055
	Wall 6	0.840–2.520		0.11–0.22
2B	Junction 1	0.420–1.260	0.840–1.260	0.055–0.11
	Junction 2	0.420–2.10		0.055–0.11
	Junction 3	0.420–1.680		0.055–0.11
	Junction 4	0.840–2.520		0.055–0.11
	Junction 5	0.840–1.260		0.00–0.11
	Junction 6	0.840–2.10		0.00–0.11
	Wall 1	0.840–1.680		0.11–0.165
	Wall 2	0.0000055–0.420		0.00–0.055
	Wall 3	0.840–1.680		0.11–0.165
	Wall 4	0.0000055–0.420		0.00–0.055
	Wall 5	0.0000055–0.420		0.00–0.055
	Wall 6	0.840–2.10		0.11–0.22
2C	Junction 1	0.420–1.680	No overlap	0.055–0.11
	Junction 2	0.840–2.520		0.055–0.11
	Junction 3	0.840–4.20		0.165–0.22
	Junction 4	1.680–3.780		0.165–0.22
	Junction 5	2.10–4.20		0.11–0.165
	Junction 6	0.840–2.940		0.11–0.165
	Wall 1	0.840–1.680		0.11–0.165
	Wall 2	0.0000055–0.420		0.00–0.055
	Wall 3	0.840–4.20		0.11–0.22
	Wall 4	1.680–2.10		0.165–0.22
	Wall 5	0.420–0.840		0.055–0.11
	Wall 6	0.0000055–1.680		0.055–0.165

## Data Availability

Data are included in the tables presented in this paper.
